# Conventional Cytogenetic Analysis of Solid Tumor Abnormalities: A 25-Year Review of Proficiency Test Results from the College of American Pathologists/American College of Medical Genetics and Genomics Cytogenetics Committee

**DOI:** 10.3390/genes15121612

**Published:** 2024-12-17

**Authors:** Rachel K. Vanderscheldon, William R. Sukov, Juli-Anne Gardner, Catherine W. Rehder, Brynn Levy, Gopalrao V. Velagaleti, Reha M. Toydemir, Guilin Tang, Brittany Boles, Yang Cao, Christopher Mixon, Ying S. Zou, Caroline Astbury, Karen D. Tsuchiya, Jess F. Peterson

**Affiliations:** 1 Department of Pathology, University of Pittsburgh, Pittsburgh, PA 15213, USA; vanderscheldenrk@upmc.edu; 2 Department of Laboratory Medicine and Pathology, Mayo Clinic, Rochester, MN 55905, USA; sukov.william@mayo.edu; 3 Department of Pathology and Laboratory Medicine, University of Vermont Medical Center, Burlington, VT 05405, USA; juli-anne.gardner@uvmhealth.org (J.-A.G.); brittney.boles@uvmhealth.org (B.B.); 4 Department of Pathology, Duke University School of Medicine, Durham, NC 27710, USA; catherine.rehder@duke.edu; 5 Department of Pathology and Cell Biology, Columbia University, New York, NY 10032, USA; bl2185@cumc.columbia.edu; 6 Department of Pathology and Laboratory Medicine, University of Texas Health Science Center at San Antonio, San Antonio, TX 78229, USA; velagaleti@uthscsa.edu; 7 Quest Diagnostics, San Juan Capistrano, CA 92675, USA; reha.m.toydemir@questdiagnostics.com; 8 Department of Hematopathology, The University of Texas M.D. Anderson Cancer Center, Houston, TX 77030, USA; gtang@mdanderson.org; 9 Department of Pathology and Immunology, Washington University School of Medicine, St. Louis, MO 63110, USA; cao.yang@wustl.edu; 10 NeoGenomics Laboratories, Nashville, TN 37211, USA; chris.mixon@neogenomics.com; 11 Department of Pathology, Johns Hopkins School of Medicine, Baltimore, MD 21205, USA; yzou19@jhmi.edu; 12 Department of Laboratory Medicine, Cleveland Clinic, Cleveland, OH 44195, USA; astburc@ccf.org; 13 The Steve and Cindy Rasmussen Institute for Genomic Medicine, Nationwide Children’s Hospital, Columbus, OH 43215, USA; karen.tsuchiya@nationwidechildrens.org; 14 Department of Pathology, Ohio State University Wexner Medical Center, Columbus, OH 43215, USA

**Keywords:** conventional chromosome analysis, solid tumors, proficiency testing, karyotype

## Abstract

*Background:* The joint College of American Pathologists/American College of Medical Genetics and Genomics Cytogenetics Committee works to ensure the competency and proficiency of clinical cytogenetic testing laboratories through proficiency testing (PT) programs for various clinical tests offered by such laboratories, including the evaluation of cytogenetic abnormalities in solid tumors. *Methods:* Review and analyze 25 years (1999–2023) of solid tumor chromosome analysis PT results, utilizing G-banded karyograms. A retrospective review of results from 1999 to 2023 was performed, identifying the challenges addressing solid tumors. The chromosomal abnormalities and overall performance were evaluated. *Results:* A total of 21 solid tumor challenges were administered during the period 1999–2018. No solid tumor challenges were administered during the period 2019–2023. Challenges consisted of metaphase images and accompanying clinical history for the evaluation of numerical and/or structural abnormalities. All 21 cases reached 80% grading consensus for abnormality recognition. However, five cases (24%) failed to reach consensus for nomenclature reporting by participating laboratories. These cases illustrate errors in reporting chromosomal abnormalities, including whole-arm translocations and those involving sex chromosomes. In addition, they highlight the challenges with differentiation of terminal and interstitial deletions, difficulties in identifying correct breakpoints, and omission of brackets in neoplastic cases. *Conclusions:* This comprehensive 25-year review demonstrates the exceptional proficiency of cytogenetic laboratories in accurately identifying chromosome abnormalities in solid tumors, while also highlighting the challenges of reporting specific types of chromosomal abnormalities.

## 1. Introduction

The value of analyzing chromosomes in solid tumor specimens became apparent as early as 1914 when Boveri observed that these neoplastic tumors often had very abnormal chromosome contents [1]. Genetic studies have played a critical, ever-growing role in the classification of solid tumors by providing insights into the molecular and chromosomal alterations that contribute to tumor development and progression. Detection and characterization of chromosomal abnormalities in solid tumors by traditional G-banded chromosome analysis can be beneficial for appropriate disease categorization, prognostication, and therapy decisions. The World Health Organization (WHO) classification of tumors continuously incorporates data on recurring chromosomal alterations into the classification of solid tumors. For example, the 2020 WHO classification introduced the subcategorization of round cell sarcomas previously described as “undifferentiated” to include the prototypical Ewing sarcoma, alongside round cell sarcomas featuring *EWSR1*-non-*ETS* fusions, *CIC*-rearranged sarcomas, and those with *BCOR* genetic variations [2]. Accurately distinguishing among these entities is vital due to differences in response to chemotherapy and overall survival rates [3]. Similarly, in Wilms tumor, the most common renal neoplasm of childhood, specific recurrent genetic changes detectable by traditional chromosome analysis are understood to impact survival [4].

Cytogenomic laboratories employ diverse methods such as fluorescence in situ hybridization (FISH), chromosomal microarray (CMA), and next-generation sequencing (NGS) to detect recurrent genetic abnormalities in solid tumors. Despite recent advancements in methodologies, traditional chromosome analysis remains an important component of diagnosing many solid tumors. Effective communication of chromosomal findings, represented in a karyotype, is critical. This communication adheres to the International System for Human Cytogenetic Nomenclature (ISCN), first published in 1978, with regular updates thereafter [5]. ISCN provides a universal framework for describing chromosomal composition, ensuring uniform reporting of abnormalities detected through techniques like G-banding, FISH, or CMA. Precise identification and documentation of abnormalities not only facilitates accurate reporting and diagnosis but also contributes to maintaining quality standards and accreditation.

Since 1986, the College of American Pathologists (CAP) and the American College of Medical Genetics and Genomics (ACMG) joint Cytogenetics Committee (CyC) (formerly Cytogenetics Resource Committee) has conducted proficiency testing (PT), ensuring precise detection and description of chromosomal abnormalities. This retrospective study, spanning 25 years (1999–2023), evaluates participant performance in identifying solid tumor chromosome abnormalities through conventional G-banding analysis. Notably, this analysis excludes assessments related to FISH and CMA programs.

## 2. Material and Methods

### 2.1. Case Selection

All conventional chromosome challenges found in the cytogenetics program (CY) from 1999 through 2023 were reviewed to identify those specifically addressing cytogenetic abnormalities in solid tumor specimens. Of note, several challenges during this time also addressed hematologic malignancies, which presented as soft tissue masses or lymph nodes. These cases were omitted from this review. Three CY program mailings (A–C, five challenges per mailing, 15 total challenges per year) were provided annually from 1999 through 2014 (240 total challenges). Two CY program mailings (A and B, six challenges per mailing, 12 total challenges per year) were provided annually from 2015 through 2023 (108 total challenges). Of the 348 total CY challenges administered between 1999 and 2023, 21 (6%) were presented in the context of a solid tumor study based on the provided clinical history and specimen type (Table 1). A designated number of metaphase cells was provided per case (paper and/or electronic images) to each enrolled cytogenetic laboratory (participants). Cases were chosen by the CyC to reflect chromosomal findings likely to be encountered during the routine conventional chromosome analysis of solid tumors.

### 2.2. Grading

From 1999 through 2008, four grading components of equal weight were utilized for each challenge, including modal chromosome number (M), sex chromosome designation (S), recognition of abnormalities (A), and karyotype nomenclature (N). In 2009, the grading components were consolidated to include the modal chromosome number, sex chromosome designation, and abnormalities under “recognition of abnormalities” (A), while maintaining “karyotype nomenclature” (N) as a separate grading component (Table 1). Grading was performed using the most current ISCN designated in the kit instructions for each challenge. Each of the four grading components (M, S, A, and N) are assigned a grade of “1” (good performance), “2” (acceptable performance), or “3” (unacceptable performance). While a score of “2” may not be the ideal response, it is technically not incorrect and still considered “acceptable”. The “modal karyotype” is defined as the most common response provided by all participating laboratories; all participant responses were graded against the modal karyotype. If at least 80% of referee laboratories (15 randomly selected laboratories from a pool of anonymized laboratories with good performance on all challenges for the previous three mailing periods) responded with the modal karyotype, or at least 80% of the participants responded with at least an acceptable karyotype, (“consensus”) the CyC formally graded the challenge; challenges that did not meet 80% consensus were not graded. In the case of subjective morphologic features, alternative ISCN forms were accepted. All participant data, as well as educational summaries written by members of the CyC, were provided to participants in a printed document, the Participant Summary Report (PSR).

## 3. Results

### 3.1. Number of Participants

The number of participants was steady overall but demonstrated an upward trajectory, ranging from a low of 208 in 2003 to a high of 318 in 2016. The upward trend from 2011 through 2018 was mainly attributable to an increase in international participation (countries outside the United States and Canada). Fifteen referee laboratories were utilized for each of the 21 challenges.

### 3.2. Specimen Types and Clinical Histories

For each of the 21 challenges, the specimen source of the metaphase images (e.g., renal tumor), in addition to a brief clinical case history was provided (Table 1). Of the 21 challenges, seven (33%) were kidney/renal tumors (including renal cell carcinoma (RCC) and Wilms tumor), four (19%) were unspecified “solid tumor” or “tumor tissue” specimens, three (14%) were soft tissue masses/tumors, three (14%) were sacral masses, one (5%) was a flank mass, one (5%) was an orbital tumor, one (5%) was a pelvic mass, and one (5%) was a retroperitoneal mass. The most common clinical information provided included patient age, tumor location (e.g., left proximal femur), and tumor type (e.g., Wilms tumor).

### 3.3. Chromosomal Abnormalities

**Figure 1:**
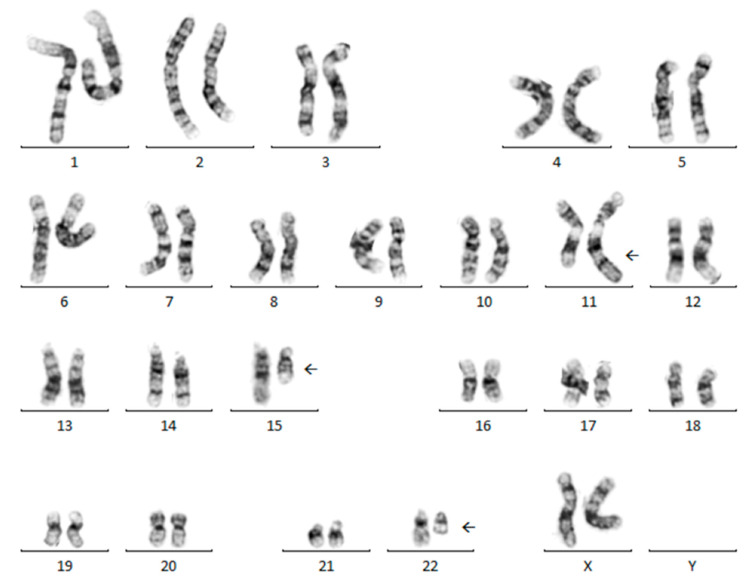
Representative metaphase cell that demonstrates t(11;22;15)(q24;q12;q15). This abnormality was challenged twice (2003B-7 and 2008A-4) and represents the variant t(11;22) that is observed in Ewing sarcoma.

None of the 21 total solid tumor challenges were “normal” (46,XX or 46,XY). Ten of 21 challenges (48%) had a single chromosomal abnormality (including interstitial deletions (2000A-1, 2003A-4, 2004C-11, 2010A-3), balanced translocations (2003B-7, 2003C-14, 2008A-4, 2016A-4, 2018A-4), and a single aneusomy (2011C-14)), eight of 21 challenges (38%) had balanced and/or unbalanced translocations and aneusomies (1999C-14, 2002B-7, 2002C-11, 2006B-8, 2010B-9, 2014A-4, 2014C-12, 2015B-12), and three of 21 challenges (14%) had multiple aneusomies with no structural abnormalities (2004A-2, 2009B-6, 2016A-2). Only one of the 21 (5%) solid tumor challenges had multiple clones (2002B-7). Several recurring chromosomal abnormalities were included in the solid tumor challenges from 1999 to 2023 (Table 1). The most common abnormality challenged was t(11;22)(q24;q12), found in Ewing sarcoma (three challenges; 2002C-11, 2003C-14, 2014A-4), with an additional variant t(11;22;15)(q24;q12;q15) (two challenges; 2003B-7, 2008A-4) (Figure 1).

### 3.4. Participant and Referee Performance

From 1999 to 2008, the modal chromosome number (M) and sex chromosome designation (S) were independently graded for each challenge. All of the modal chromosome number and sex chromosome designation components (referee or participants) met the ≥80% grading threshold for the period 1999–2008.

Recognition of abnormalities (A) and karyotype nomenclature (N) components were graded independently for the period 1999–2023. All 21 challenges met the 80% consensus for abnormality identification by both referees and participants (Table 1). However, five of 21 challenges (24%) failed to meet the 80% consensus for karyotype nomenclature by the participants (1999C-14, 2000A-1, 2002B-7, 2003A-4, 2006B-8). Of those five challenges, two had 11p deletions as the sole abnormalities (2000A-1, 2003A-4), two had a t(X;18)(p11.2;q11.2) and trisomy 17 (1999C-14) and the other with multiple aneusomies and a subclone (2002B-7), and one had an unbalanced t(3;8) (2006B-8) (Table 1).

**Figure 2:**
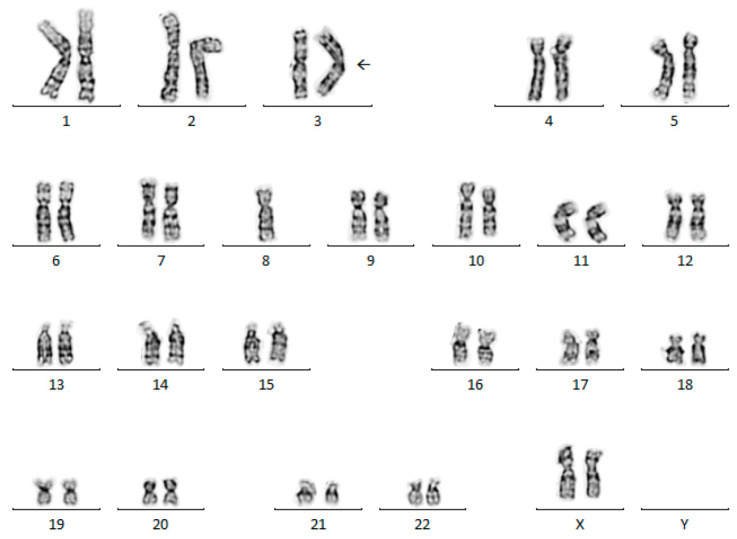
Representative metaphase cell from challenge 2006B-8. This challenge failed to meet 80% consensus for karyotype nomenclature. Three karyotypes were acceptable: 45,XX,der(3;8)(q10;q10)[5], 45,XX,der(3)t(3;8)(p11;q11.1),-8[5], and 45,XX,-3,der(8)t(3;8)(q11.1;p11.1)[5]. Most participants correctly identified and described a whole-arm translocation.

Of the 21 total cases, 18 (86%) had a single acceptable karyotype while three (14%) had multiple ISCN designations that were deemed acceptable by the CyC (2002C-11, 2006B-8, 2015B-12) (Table 1). For challenge 2002C-11, both “der(16)t(1;16)(q21;q13)” and “+1,der(1;16)(q10;p10)” were deemed acceptable in the karyotype nomenclature. Similarly, “der(3;8)(q10;q10)”, “der(3)t(3;8)(p11;q11.1),-8”, and “-3,der(8)t(3;8) (q11.1;p11.1)” were deemed acceptable in the karyotype nomenclature for challenge 2006B-8 (Figure 2). Lastly, two acceptable karyotypes were accepted for 2015B-12, including “t(4;19)(q35;q13.1)” and “t(4;19)(q35;q13.2)”, each with different breakpoints for the derivative chromosome 19 (q13.1 vs q13.2).

## 4. Discussion

Throughout the 25-year review period (1999–2023), the number of laboratories participating in the solid tumor CyC PT program increased, with 250 participating laboratories in 1999, a peak of 318 participating laboratories in 2016, and 303 participating laboratories in 2018. Historically, conventional chromosome analysis has predominantly been utilized for constitutional disorders and hematologic neoplasms due to the relative ease of collecting metaphase cells for analysis. This has propelled significant advancements in understanding the genetic pathology in these fields, while progress in lymphomas and solid tumors has been comparatively slower due to the challenges of obtaining dividing cells from these types of neoplasms [6]. Conventional karyotyping requires fresh specimens and often leads to normal karyotypes due to the overgrowth of normal cells. Additionally, the low resolution often hinders precise characterization of gene rearrangements [7]. Next-generation sequencing (NGS) and RNA-based gene fusion assays, as well as CMA, have overcome these limitations by offering higher resolution and precision in identifying gene rearrangements and unbalanced copy number abnormalities, respectively. This has led to the replacement of karyotyping with NGS-based assays and CMA in many clinical laboratories, enabling tailored treatment options based on genomic abnormalities. However, while traditional chromosome analysis for solid tumors may be declining in various laboratories due to the rising preference for FISH, molecular, and/or NGS, these advanced methods may not be accessible in certain developing regions. In addition, conventional chromosome analysis may be the most practical methodology utilized to evaluate solid tumor clones in smaller cytogenomic laboratories that may not have robust FISH and/or NGS-based test menus. Lastly, a renewed interest in conventional cytogenetics and its combinatory potential with cytogenomics has been recently described. Studies recognize chromosome instability as a driving force in cancer evolution, with karyotype heterogeneity serving as an important biomarker [8,9].

PT relies heavily on the availability of samples from vendors; as more and more laboratories move away from conventional chromosome analysis to utilize CMA, molecular studies, and RNA-based fusion assays for solid tumors, the number of samples available for testing has declined. This reality has led to a divergent number of solid tumor cases presented in the CY proficiency program compared to constitutional and hematological cases. Between 1999 and 2023, a total of 21 solid tumor cases were challenged. In comparison, other published summaries of the CY PT program showed that 184 constitutional cases were provided between 2003 and 2022 and 288 hematologic neoplasm cases were provided between 1999 and 2018 [10,11].

The 15 referee laboratories for all 21 solid tumor cases met the 80% threshold for both abnormality identification and karyotype nomenclature, therefore all 21 of the solid tumor challenges were graded. Impressively, participating laboratories scored above 80% for abnormality recognition for each of the 21 cases, while in five cases (24%) they scored below 80% for karyotype nomenclature.

Two cases that scored below 80% for karyotype nomenclature had 11p deletions in a Wilms tumor (2000A-1 and 2003A-4). Case 2000A-1 was presented as a renal tumor in a 2-year-old with aniridia, with the intended karyotype of 46,XX,del(11)(p11.2p13)[5]. The most common error in nomenclature, observed in two of fifteen (13%) referee responses and 51 of 252 (20%) participant responses, was the omission of brackets indicating the number of abnormal cells. Given the clinical history of aniridia, it is likely that many laboratories assessed this to be a constitutional abnormality resulting in Wilms tumor with aniridia, genitourinary abnormalities, and a range of developmental delays (“WAGR” syndrome) [12]. This interpretation may explain the frequent omission of brackets, which are required for all neoplastic cases. However, only two laboratories included “c” after the karyotype designation, which would indicate a constitutional abnormality. The PSR provided participant education. In addition, several interpretive questions were included in the 2001 CY-A mailing (not included in Table 1), including a question regarding the peripheral blood findings in a sample from a child with aniridia and Wilms tumor. The educational material emphasized the importance of omitting a semicolon between breakpoints in a single chromosome rearrangement. The second case challenging Wilms tumor (2003A-4) was presented as a retroperitoneal mass in a 5-year-old, and the intended karyotype was 46,XX,del(11)(p11.2p14)[5]. The most common error was incorrect identification of the abnormality as a terminal deletion, observed in 32 of 208 (15%) participant responses. Since the intended abnormality was an interstitial deletion of the short arm of chromosome 11, identification as a terminal deletion was considered acceptable for recognition of abnormality but unacceptable for karyotype nomenclature. Deletions of 11p are relatively rare, found in approximately 10–20% of Wilms tumors [4]. Therefore, while additional clinical features of WAGR syndrome were not provided in the clinical history, 22 of 208 (11%) participating laboratories still omitted brackets indicating the number of abnormal metaphases. In this case, no laboratories included the constitutional “c” nomenclature, perhaps demonstrating the effectiveness of educational materials in prior PSRs. If these challenges were given today, the correct nomenclature would be 46,XX,del(11)(p13p11.2)[5] and 46,XX,del(11)(p14p11.2)[5], respectively, as ISCN 2020 specifies that breakpoints should be listed from the terminus towards centromere.

Two additional cases that did not reach the 80% participant consensus for karyotype nomenclature (1999C-14 and 2002B-7) challenged the identification of t(X;18), a hallmark of synovial sarcoma. For 1999C-14, the intended karyotype was 47,X,t(X;18)(p11.2;q11.2),+17[5]. Again, the most common error of nomenclature was the omission of brackets, seen in 40 (16%) cases. A challenging aspect of this case was the proximity of breakpoints to the centromeres, leading 30 (12%) participant laboratories to interpret this case as an X;18 whole-arm translocation. This interpretation was accepted if breakpoints were appropriately assigned. In case 2002B-7, the intended karyotype was 51,Y,t(X;18)(p11.2;q11.2),+2,+6,+12, +16,+17[3]/52,idem,+4[2]. Like case 1999C-14, some laboratories interpreted this as a whole-arm translocation, and the CyC accepted this response. However, this complex karyotype posed additional challenges. Twenty-three of 250 laboratories (9%) correctly identified the sex chromosomes but misplaced them within the karyotype. According to ISCN 1995 guidelines, for karyotypes in which a translocation affects one of the sex chromosomes, the normal sex chromosome should be listed first.

The final case not meeting 80% consensus for karyotype nomenclature in the participant group was 2006B-8, which involved a kidney tumor with a whole-arm translocation (Figure 2). Three karyotypes were acceptable: 45,XX,der(3;8)(q10;q10)[5], 45,XX,der(3)t(3;8)(p11;q11.1),-8[5], and 45,XX,-3,der(8)t(3;8)(q11.1;p11.1)[5], and most participants correctly identified and described a whole-arm translocation. In this case, the most common errors involved reporting breakpoints outside of the acceptable range.

A three-way translocation, including the t(11;22) found in Ewing sarcoma, was challenged twice (2003B-7 and 2008A-4). Recognition of abnormality in the participant group remained excellent in both cases, reaching 94% and 96%, respectively. Correct use of nomenclature in the participant group improved between the two challenges, reaching 83% in the 2003 challenge and increasing to 94% in the 2008 challenge, perhaps due to educational material in the PSR.

The CyC accepted multiple karyotype designations for three cases, including 2002C-11, 2006B-8 (discussed above), and 2015B-12. For case 2002C-11, the intended karyotype was 48,XY,+8,t(11;22)(q24;q12),+12,der(16)t(1;16)(q21;q13)[5]. However, the interpretation of the rearrangement involving chromosomes 1 and 16 as a whole-arm translocation resulted in alternative karyotype nomenclature. Thus, the following nomenclature was also accepted: 48,XY,+1,der(1;16)(q10;p10),+8,t(11;22)(q24;q12),+12[5]. For case 2015B-12, limited band-level resolution of the metaphases prevented conclusive characterization of the breakpoints associated with the abnormalities.

In general, several recurring errors were observed across many of the reviewed solid tumor cases. For example, ISCN guidelines require that the number of both neoplastic and normal metaphases should be listed in brackets. Despite addressing this requirement in the Grading Criteria section of the Kit Instructions and several PSRs, this was a recurrent error. In addition, cases featuring distal interstitial deletions were often mischaracterized as terminal deletions, highlighting limitations in banding resolution. Cases featuring whole-arm translocations also continuously posed a challenge to participants.

In summary, ongoing assessment in the form of PT is an important component of education for laboratories conducting conventional chromosome analysis for solid tumors. From 1999 to 2023, laboratory participation in CyC programs fluctuated but remained overall high. Over this period, the CyC administered 21 challenges featuring common and clinically important cytogenetic abnormalities occurring in solid tumors. The consistent identification of recurrent errors, especially concerning karyotype nomenclature, highlights areas for continued improvement within the cytogenetics community and underscores the need for targeted education in future Participant Summary Reports.

## Figures and Tables

**Table 1:** Performance summary of a 25-year retrospective review (1999 through 2023) of karyotype challenges specifically addressing solid tumor abnormalities.

Year	CY Survey	Specimen #	Specimen	Case History	Accepted Karyotype(s)	Diagnosis and/or Discussion Point(s)	Referees			Participants		
							#	A, Acceptable (%)	N, Acceptable (%)	#	A, Acceptable (%)	N, Acceptable (%)
1999	C	14	Soft tissue mass	Soft tissue mass in the calf of a 7-year-old	47,X,t(X;18)(p11.2;q11.2),+17[5]	Synovial sarcoma characterized by t(X;18)	15	15 (100)	15 (100)	250	228 (91)	**174 (70)**
2000	A	1	Renal tumor	Renal tumor of a 2-year-old with aniridia	46,XX,del(11)(p11.2p13)[5]	Discovery of 11p13 deletions in patients with the aniridia–Wilms tumor association	15	15 (100)	13 (87)	252	226 (90)	**173 (69)**
2002	B	7	Soft tissue mass	Soft tissue mass from a 25-year-old with a 6-month history of an enlarging mass in the right calf	51,Y,t(X;18)(p11.2;q11.2),+2,+6,+12,+16,+17[3]/52,idem,+4[2]	t(X;18) observed in synovial sarcoma	15	13 (87)	12 (80)	250	227 (91)	**188 (75)**
	C	11	Sacral mass	Fine needle aspirate of a sacral mass	48,XY,+8,t(11;22)(q24;q12),+12,der(16)t(1;16)(q21;q13)[5] OR 48,XY,+1,der(1;16)(q10;p10),+8,t(11;22)(q24;q12),+12[5]	Ewing sarcoma family of tumors associated with t(11;22)(q24;q12)	15	15 (100)	14 (93)	244	229 (94)	210 (86)
2003	A	4	Retroperitoneal mass	Retroperitoneal mass from a 5-year-old	46,XX,del(11)(p11.2p14)[5]	Deletions in 11p occur in 20% of Wilms tumor, the most common pediatric cancer of the kidney	15	15 (100)	14 (93)	208	206 (99)	**154 (74)**
	B	7	Sacral mass	Excisional biopsy of a sacral mass in a 13-year-old	46,XX,t(11;22;15)(q24;q12;q15)[5]	Variant of the t(11;22) that is well known in Ewing sarcoma and related tumors	15	15 (100)	15 (100)	243	229 (94)	202 (83)
	C	14	Flank mass	Left flank mass of an 18-year-old	46,XX,t(11;22)(q24;q12)[5]	t(11;22) characteristic of the Ewing sarcoma family of tumors	15	15 (100)	15 (100)	237	230 (97)	219 (92)
2004	A	2	RCC	54-year-old male referred for RCC	50,X,-Y,+3,+7,+12,+16,+17[5]	Common recurring abnormalities in papillary RCC are +7, +17 and loss of the Y chromosome	15	14 (93)	14 (93)	212	200 (94)	196 (92)
	C	11	RCC	Solid tumor of a patient referred for RCC	46,XX,del(3)(p13p25)[5]	Clear cell RCC is characterized by deletions of 3p, most frequently involving loss of 3p12-14, 3p21, and 3p25	15	14 (93)	14 (93	236	234 (99)	228 (97)
2006	B	8	Kidney tumor	Kidney tumor from a 58-year-old	45,XX,der(3;8)(q10;q10)[5] OR 45,XX,der(3)t(3;8)(p11;q11.1),-8[5] OR 45,XX,-3,der(8)t(3;8)(q11.1;p11.1)[5]	A 3p deletion and loss of chromosome 8 are most common in adenoma and carcinoma of the proximal tubule	15	13 (87)	13 (87)	213	185 (87)	**167 (78)**
2008	A	4	Sacral mass	Excisional biopsy of a sacral mass in a 13-year-old	46,XX,t(11;22;15)(q24;q12;q15)[5]	Three-way translocation includes the t(11;22) that is found in the majority of Ewing sarcoma/pripheral (primitive) neuroectodermal tumors	15	15 (100)	15 (100)	221	212 (96)	207 (94)
2009	B	6	Solid tumor	Solid tumor from an 80-year-old male referred for questionable RCC	51,X,-Y,+3,+7,+7,+16,+17,+20[5]	Hyperdiploid karyotypes with loss of a sex chromosome and the pattern of chromosomal gains are well documented recurring abnormalities in papillary RCC	15	15 (100)	15 (100)	241	237 (98)	235 (98)
2010	A	3	Solid tumor	Solid tumor excised from an 8-month-old	46,XY,del(13)(q14.1q22)[5]	Deletion of 13q associated with retinoblastoma	15	15 (100)	15 (100)	254	251 (99)	248 (98)
	B	9	Solid tumor	Solid tumor resected from the right thigh of a 40-year-old	48,XY,+5,+8,t(12;16)(q13;p11.2)[5]	t(12;16)(q13;p11.2) associated with myxoid liposarcoma	15	15 (100)	15 (100)	282	263 (93)	253 (90)
2011	C	14	Renal tumor	Renal tumor removed from a 63-year-old	47,XX,+7[5]	Association of trisomy 7 with RCC	15	15 (100)	15 (100)	314	313 (99)	304 (97)
2014	A	4	Tumor tissue	Tumor tissue specimen from the left proximal femur of a 10-year-old	50,XY,+5,+8,+8,t(11;22)(q24;q12),+15[5]	Approximately 90% of Ewing sarcoma/primitive neuroectodermal tumors show the t(11;22)	15	14 (93)	13 (87)	313	264 (84)	255 (82)
	C	12	Wilms tumor	A 5-year-old with Wilms tumor	45,XX,+1,der(1;16)(q10;p10),-10,der(22)t(10;22)(q11.2;q13)[5]	Gain of 1q, loss of 16q, and loss of all or part of 22q have been associated with an adverse outcome in patients with Wilms tumor	15	15 (100)	14 (93)	274	256 (93)	222 (81)
2015	B	12	Pelvic mass	Left pelvic mass from a 20-year-old who had a normal constitutional karyotype	45,X,-X,t(4;19)(q35;q13.1)[3]/46,XX[2] OR 45,X,-X,t(4;19)(q35;q13.2)[3]/46,XX[2]	t(4;19) is a recurrent finding in pediatric and adult undifferentiated round cell sarcoma	15	14 (93)	13 (87)	308	263 (85)	248 (81)
2016	A	2	RCC	RCC from an 80-year-old male	51,X,-Y,+3,+7,+7,+16,+17,+20[5]	Abnormalities consistent with the diagnosis of papillary RCC	15	15 (100)	15 (100)	318	316 (99)	311 (98)
		4	Orbital tumor	Left orbital tumor from a 13-year-old referred for malignant neoplasm/sarcoma	46,X,t(X;17)(p11.2;q25)[5]	t(X;17) has been reported in alveolar soft part sarcoma, including those of the orbit, and some cases of RCC	15	15 (100)	15 (100)	317	300 (95)	288 (91)
2018	A	4	Soft tissue tumor	Lower extremity soft tissue tumor from a 14-year-old	46,XX,t(4;19)(q35;q13.1)[5]	A rare subset of undifferentiated small round cell sarcomas with t(4;19)(q35;q13.1) have distinctive and reproducible morphologic features	15	15 (100)	15 (100)	303	286 (94)	277 (91)

A, abnormality recognition; N, karyotype nomenclature; RCC, renal cell carcinoma; Bolded data indicate karyotype nomenclature that did not meet 80% consensus for participant groups.

## Data Availability

All data and materials are available individually upon request to the corresponding author.
